# Global perspectives on rheumatology training: insights from the EULAR 2025 EMEUNET Presents session

**DOI:** 10.1016/j.ero.2026.02.005

**Published:** 2026-03-12

**Authors:** Latika Gupta, Alexandros Panagiotopoulos, Dzifa Dey, Wilson Bautista-Molano, Sunith Himantha Atukorale, Ibtissam Gad, Diego Benavent, Tue Wenzel Kragstrup, Alessia Alunno

**Affiliations:** 1 Department of Rheumatology, Royal Wolverhampton Hospitals NHS Trust, Wolverhampton, UK; 2 School of Infection, Inflammation and Immunology, College of Medicine and Health, University of Birmingham, Birmingham, UK; 3 Francis Crick Institute, London, UK; 4 Rheumatology Unit, First Department of Propaedeutic Internal Medicine, Laiko Hospital, Medical School, National & Kapodistrian University of Athens, Athens, Greece; 5 Rheumatology Unit, Department of Medicine and Therapeutics, University of Ghana Medical School, College of Health Sciences, Accra, Ghana; 6 Rheumatology Department, University Hospital Fundación Santa Fe de Bogotá, School of Medicine Universidad El Bosque, Bogotá, Colombia; 7 Department of Rheumatology, District General Hospital Gampaha, Ministry of Health, Colombo, Sri Lanka; 8 Department of Internal Medicine, Division of Rheumatology, University of Michigan Medical School, Ann Arbor, MI, USA; 9 Department of Rheumatology, IDIBELL-Hospital Universitari de Bellvitge, University of Barcelona, Barcelona, Spain; 10 Department of Biomedicine, Aarhus University, Aarhus, Denmark; 11 Department of Molecular Medicine, Aarhus University Hospital, Aarhus, Denmark; 12 Rheumatology Sector, Medical Diagnostic Centre, Silkeborg Regional Hospital, Silkeborg, Denmark; 13 Internal Medicine and Nephrology Division, Department of Clinical Medicine, Life, Health and Environmental Sciences (MeSVA), University of L’Aquila, L’Aquila, Italy

## Abstract

**Objectives:**

Global rheumatology training faces immense challenges, with over 1.7 billion people affected by musculoskeletal diseases and pronounced regional disparities in specialist access. Diverse curricula, resource limitations, and variable career pathways underscore the urgency for targeted harmonisation efforts. This study aims to present a vision for harmonising rheumatology training worldwide, highlighting the contributions of continental societies and innovative young networks, based on insights from the EMerging European Alliance of Associations for Rheumatology (EULAR) NETwork (EMEUNET) Presents session at EULAR Congress 2025 ‘Rheumatology Training Around the Globe’.

**Methods:**

A narrative review of current training models across Africa, Asia-Pacific, Pan-America, and Europe using data from EULAR 2025 Congress supplemented with session slides and speaker insights.

**Results:**

Programmes in Africa remain limited and fragmented, with severe workforce shortages and few structured training opportunities; regional responses include Young African Rheumatologists Network and select international partnerships. Asia-Pacific features multicountry, exam-based curricula governed centrally, but faces shortfalls in infrastructure and fellowship access, addressed in part by Asia Pacific League of Associations for Rheumatology-Young Rheumatology’s educational initiatives. Pan-America is marked by significant programme heterogeneity and access barriers, with the United States standing out for its highly structured American College of Rheumatology-accredited programmes, robust educational events, and comprehensive resources. Pan-American League of Associations for Rheumatology Joven and digital education initiatives are reducing training gaps and advancing collaboration. Europe combines robust, competency-focused national programmes, with EULAR and European Union of Medical Specialties actively supporting harmonisation despite persistent local variation, and EMEUNET enhancing mentorship and career support.

**Conclusions:**

Future harmonisation in rheumatology training requires flexible, competency-driven frameworks, expanded digital platforms, multilevel mentorship, and sustainable investment, integrating national programmes into global networks.

## INTRODUCTION

Rheumatology faces an unprecedented challenge: over 1.7 billion people worldwide suffer from musculoskeletal diseases [1], yet specialist availability varies dramatically across regions. Global rheumatology training presents a complex mosaic, shaped by local healthcare systems, economic realities, and cultural contexts. European programmes typically require 4 to 6 years with structured curricula, whereas Latin American pathways involve 2 to 3 years following internal medicine training. The Asia-Pacific region demonstrates remarkable diversity, from highly developed programmes to emerging initiatives in resource-limited settings [2].

Although some countries maintain robust training programmes with comprehensive resources, others struggle with fundamental shortages that leave populations without specialist care [3]. This disparity represents more than an equity issue; it threatens global health security and limits our collective ability to advance the specialty. The session ‘Rheumatology Training Around the Globe’ held at the 2025 Annual Congress of the European Alliance of Associations for Rheumatology (EULAR) revealed both sobering realities and inspiring innovations in addressing these challenges.

Major training frameworks worldwide share common structural elements, including didactic education, clinical rotations, research components, and competence assessments. Whether inspired by training requirements of overarching institutions such as the American Accreditation Council for Graduate Medical Education, or national/regional adaptations, these frameworks demonstrate convergence in core educational objectives and assessment strategies.

However, this apparent consistency masks significant variations in implementation, resource availability, and educational outcomes. Although formal curricula may appear harmonised, training delivery varies considerably based on local factors, including faculty expertise, patient populations, healthcare infrastructure, and institutional resources.

Representatives from 4 continental leagues shared experiences and the unmet need to learn from experiences and work towards the aspirational goal of a more harmonised, equitable global training ecosystem. Their collective insights suggest that the future of rheumatology training lies not only in isolated national programmes but also in interconnected networks that leverage diverse strengths while maintaining rigorous standards. The following excerpts feature a narrative synthesis from EULAR 2025 Congress speaker slides and session discussion insights on current training models across 4 continental regions (Africa, Asia-Pacific, Pan-America, and Europe).

## AFRICAN RHEUMATOLOGY TRAINING: CRITICAL SHORTAGE AND EMERGING SOLUTIONS

Africa faces a severe shortage of the rheumatology workforce. Recent estimates suggest that there are approximately 0.25 rheumatologists per 100,000 people across Africa, with 17 countries having no rheumatologists and only 64 rheumatologists serving Nigeria’s population of over 180 million as of 2022 [4]. Training opportunities remain concentrated in a few countries and capitals, such as North Africa; many regions lack centres for training, teaching specialists, protected time, and access to core diagnostics (autoantibody testing, ultrasound) and advanced therapies, such as biologics. Early-career clinicians often rely on ad-hoc mentorship rather than structured rotations, limited and high cost for funding training, health worker migration to higher-income countries, and minimal undergraduate rheumatology exposure when making career choices [5,6].

The increasing prevalence and recognition of rheumatic diseases across African countries further highlight the urgent need for more specialist rheumatologists [7]. Africa’s rheumatology training landscape is expanding, but capacity still lags behind the need. A handful of structured, accredited pathways anchor growth, complemented by continental webinar series and virtual programmes that form the scaffolding for a stronger, more equitable workforce to meet the continent’s needs. Regional disparities highlight the need to scale context-appropriate education and leadership development across Anglophone, Francophone, and Lusophone Africa.

Despite these constraints, Africa has the components of a training ecosystem, including structured fellowships in North Africa, South Africa, Nigeria, Ghana, and some Francophone countries, continent-wide harmonised curricula, and tested virtual programmes. The next step is to connect and expand these components, for example, by creating regional hubs that host rotations and formalise ‘centres of excellence’ that host short observerships and 6-12 months rotations for trainees from neighbouring countries, leveraging South African, West African College of Physicians and Ghana College of Physicians and Surgeons frameworks to recognise time spent, assess competencies, standardise and harmonise curricula, establish seed grants for South-South training, and develop metrics that incentivise retention and service expansion. Additional detail on country-specific programmes is provided in Table and Supplementary Material S1.

**Table:** Comparative analysis of rheumatology training parameters across global regions

Training metrics	Africa	Asia-Pacific	Pan-America	Europe/US
Total rheumatology training duration (post-MD)	2-4 y^a^	5-6 y^a^^,^^b^	4-6 y^a^	5-6 y (US); 4-6 y (Europe)^a^
Prerequisite core training (internal medicine)	Variable	3-4 y^a^^,^^b^	2-3 y^a^	2-3 y (US, Europe^a^)
Training centres	Concentrated in few countries (South Africa, Nigeria, Ghana, North Africa)	17/18 countries; majority with multiple centers^b^	Highly heterogeneous; major disparities	Widely distributed; standardised in many countries
Curriculum structure	Mix of structured and ad-hoc; WACP and GCPS standardised tracks	Centralised governance; exam-based; diverse formats	Nonstandardised; variable requirements	Standardised national/regional programmes; competency-based frameworks
Assessment methods	Clinical + written exams (where available)	Rigorous, clinical + written exams; board certification in 17/18 countries^b^	Limited standardisation; variable assessment	Standardised exams; EULAR competency frameworks; ACGME domains (US)
Accreditation	Limited; emerging structures	Mandatory in 14/18 countries^b^	Variable; many countries lack formal accreditation	Mandatory; EULAR-UEMS standards; ACGME accreditation (US)
Funding sources	Limited; often trainee-dependent; few government-supported programmes	Government (11/18); training hospitals (6); universities (3)^b^	Mixed; often limited government support	Government-institutional supported
Young rheumatologist networks	YARN (emerging)	AYR (established; large community)	PANLAR Joven (mature; innovative virtual)	ACR-FIT (US)
EMEUNET (EULAR) (established; multicountry; mentorship-focused)				
Digital/virtual training	Emerging (RFA, EULAR–AFLAR courses, WhatsApp networks)	Limited but expanding	Advanced (virtual journal clubs, Copa América competitions)	Established (online courses, webinars, digital platforms)
Research integration	Minimal in most programmes	Mixed; dissertation requirements in some; limited access to research	Limited protected time; clinical focus predominates	Protected time; integration in curricula; EULAR research initiatives

ACGME, Accreditation Council for Graduate Medical Education; ACR-FIT, American College of Rheumatology-Fellows in Training; AFLAR, African League of Associations for Rheumatology; APLAR, Asia Pacific League of Associations for Rheumatology; AYR, APLAR Young Rheumatologists; EMEUNET, Emerging EULAR NETwork; EULAR, European Alliance of Associations for Rheumatology; GCPS, Ghana College of Physicians; MD, doctor of medicine; PANLAR, Pan-American League of Associations for Rheumatology; RFA, Rheumatology For All; UEMS, European Union of Medical Specialties; US, United States; WACP, West African College of Physicians; YARN, Young African Rheumatologists Network.

a Variable by country.

b Unpublished data from the AYR (APLAR Young Rheumatologists) member survey.

Catalytic partnerships, such as those offered by Rheumatology For All (RFA), which funds local physician rheumatology training and runs virtual lecture series in Ethiopia and Rwanda, have led to the training of the first rheumatologist in Ethiopia and the training of rheumatologists and internists in Rwanda [8]. The Understanding and Working to Enhance outcomes in Zoetic (Organopedic/Health) education (UWEZO) programme in Kenya is another example of a successful international partnership focused on musculoskeletal health training [9]. To build resources for training, some continental initiatives have emerged, such as the African League of Associations for Rheumatology (AFLAR)’s education arm lecture series (AFLAR-Training, Education, and African Collaboration in Health) and the EULAR–AFLAR initiative, which provides free access to high-quality online rheumatology courses for clinicians in sub-Saharan Africa, is an important bridge where local resources are few [6].

AFLAR’s newly formed Young African Rheumatologists Network (YARN) aims to coordinate a collective vision and efforts, targeting various grants and exchange programmes by 2025 [10]. Innovation is emerging as a result of digital solutions. EULAR Online Courses, visiting faculty programmes, webinars, and WhatsApp groups that overcome geographical barriers. By connecting emerging rheumatologists, building skills, and advocating for the specialty, YARN embodies the collaborative approach needed to address Africa’s rheumatology training crisis and build sustainable specialised care across the continent.

## ASIA-PACIFIC RHEUMATOLOGY TRAINING: STRUCTURED DIVERSITY AND ESTABLISHED NETWORKS

The Asia-Pacific region demonstrates a more structured approach to rheumatology training, with comprehensive survey data from 19 Asia Pacific League of Associations for Rheumatology (APLAR) member countries revealing both diversity and standardisation [11]. Nearly all countries (18/19) have official rheumatology training schemes, although some trainees still seek international training in the United Kingdom, United States (US), Canada, Ireland, Bangladesh, and India.

The training structures vary significantly across regions. Ten countries operate single national schemes, and 6 maintain multiple schemes across regions or institutions. The remainder use defined basic curricula modified by individual institutions to meet their needs. Strong governance exists, with 15 of 18 countries having central governing bodies ranging from national rheumatology training committees (Malaysia) to ministries of health (Kazakhstan and Iran) and professional colleges (Japan College of Rheumatology and Hong Kong College of Physicians). Additional details on programme descriptions are available in Table and Supplementary Material S2.

Despite this structure, significant challenges persist, including limited training centres, low stipends, competitive fellowship slots, rapidly evolving therapies, and inadequate infrastructure. Limited access to biologics and research opportunities limits investment in the pharmaceutical industry, thus further compounding these issues.

The APLAR Young Rheumatologists (AYR) network [12], established for rheumatologists aged ≤45 years, provides structured support through educational webinars, research grants, and travel funding to provide collateral exposure to young rheumatologists and catalyse international collaboration [13]. With extensive ambassador networks and evolving active partnerships via the AYR partnerships committee with organisations like AFLAR, AYR represents a rapidly maturing model for young rheumatologist development and regional collaboration.

## PANAMERICAN RHEUMATOLOGY TRAINING: BRIDGING GAPS THROUGH INNOVATION

The Panamerican region presents a complex landscape of rheumatology training, marked by significant disparities. Unlike the more structured Asia-Pacific model or the resource-constrained African scenario, Latin America demonstrates an unequal distribution and heterogeneity of training programmes across the different countries, creating substantial access barriers for aspiring rheumatologists [14].

The training of rheumatologists across Latin America remains highly heterogeneous, reflecting differences in healthcare systems, institutional resources, and local priorities (additional details in Table, Supplementary Material S3). In several countries, rheumatology is considered a subspecialty with limited training positions, which creates disparities in access to formal education and mentorship. This uneven distribution of programmes results in substantial gaps in workforce capacity, with some regions experiencing a critical shortage of specialists to meet the growing burden of rheumatic and musculoskeletal diseases on the continent.

Despite these challenges, the region has shown promising innovations. The growing use of virtual education and telemedicine has expanded access to specialised training [15], while the increasing involvement of young rheumatologists in leadership roles and academic exchanges is driving positive change. The region benefits from a shared language and cultural connections that facilitate cross-border collaboration and further potential uniformity.

Pan-American League of Associations for Rheumatology (PANLAR) Joven (Young PANLAR) represents a mature model for young rheumatologist development, offering virtual journal clubs, monthly case discussions, and the innovative ‘PANLAR Copa América’ interactive knowledge competition [[16], [17], [18]]. The organisation facilitates research collaborations and symposiums with international academic societies while maintaining active social media outreach and advocacy for young professionals. This strategy represents a pivotal effort to strengthen rheumatology education and clinical expertise across Latin America. By fostering standardised training curricula, promoting exchange programmes (eg, fellowships), and integrating mentorship opportunities, this effort aims to reduce disparities in access to specialised care and training with the objective to build stronger and more connected rheumatology communities across the Americas and to enhance the quality of musculoskeletal health services in the region.

International partnerships demonstrate the region’s commitment to global collaboration. Joint initiatives with the Emerging EULAR NETwork (EMEUNET) and APLAR create cross-continental learning opportunities, exemplified by collaborative webinars addressing ‘Rheumatology in Resource-Limited Settings’ and specialised symposiums on psoriatic arthritis and difficult-to-treat conditions [19]. The experience of training abroad highlights both the value of international exposure and the brain-drain challenges facing the region. However, many trainees return to contribute to local programmes, bringing a global perspective to regional training.

## UNITED STATES RHEUMATOLOGY TRAINING: STRUCTURED EXCELLENCE AND INNOVATION

The US maintains a highly structured and competitive rheumatology training system, with 132 adult rheumatology programmes achieving exceptional fill rates of 99% in the 2024 Match (284 of 287 positions filled from 381 applicants; additional details in Table, Supplementary Material S4). Despite this well-rounded structure, challenges persist, including the rheumatology workforce shortage in the US, which is attributed to an ageing population, uneven geographical distribution of rheumatologists, limited training positions, and increased demand for part-time positions [20]. Other challenges include student debt, balancing research and clinical training, supporting trainees through major life transitions like parenthood, strategies to reduce burnout, and addressing work-life balance during intensive fellowship years [21,22].

The American College of Rheumatology (ACR) Fellows-in-Training (FIT) Subcommittee supports fellows by organising programming at national conferences, including scientific sessions, networking, and career planning. Recently, the FIT subcommittee introduced innovative educational activities like ‘Picture This’ clinical image challenges, and ‘The Great Debate’ competitions to increase engagement using active learning strategies [23]. The FIT subcommittee further supports fellows by sharing ACR’s robust resources including board preparation resources, mentorship programmes (ACR/Childhood Arthritis Research and Rheumatology Alliance Mentoring Interest Group/Creating Adult Rheumatology Mentorship in Academia), career planning tools, scholarships, and grant funding. The ACR as an organisation has also advanced fellow learning by providing opportunities to participate as volunteers in rheumatology guideline development and advocacy. The rheumatology medical education community has vested much discussion and research to further improve resources and curricula for trainees [24].

The system’s strength lies in its emphasis on mentorship, comprehensive resource availability, and strong professional networking, exemplified by collaborative international initiatives that position US fellows as global leaders in rheumatology education and practice. Innovation continues through digital platforms, virtual learning tools, and enhanced international collaboration, ensuring that US rheumatology training adapts to evolving global healthcare needs while maintaining its position as a world-class training destination.

## POSTGRADUATE TRAINING IN THE EULAR AREA: JOINING FORCES TO DEVELOP HARMONISATION STRATEGIES

Postgraduate rheumatology training is offered in 41 of the 45 EULAR countries, with programmes established at the national level [25]. A mapping exercise conducted by EULAR over the last decade highlighted significant differences in the learning structure and assessment of competences among European countries as well as regional/local differences in the implementation of the national programmes (Table, Supplementary Material S5). Despite this, great heterogeneity might be expected to lead to education inequalities across Europe, competences seem to be achieved irrespective of that, supporting the high quality and rigour of individual training programmes [25,26].

Within this region, exemplary national training programmes have been established in the Nordic countries, the Netherlands, and the United Kingdom, which have implemented rigorous, competency-focused curricula and robust assessment frameworks that can serve as models for harmonisation efforts. The EULAR work on the new examination and the standards developed in collaboration with the European Union of Medical Specialties (UEMS) are explicitly aimed at harmonising training and represent exemplary models of how this can be accomplished. A systematic literature review and focus groups with European trainees and trainers informed the 2019 EULAR points to consider for the assessment of competences in rheumatology specialty training, which covered overall assessment strategies, formative assessment, assessment of knowledge, skills or professionalism, and training of trainers [[27], [28], [29]]. In line with this, and by collating the experience and documents developed at national level, an EULAR portfolio and assessment forms were also developed [30].

Building on this earlier work, the 2023 points to consider: EULAR-UEMS standards for the training of European rheumatologists were developed with the overarching aim of defining core competences required to qualify as a trained rheumatologist and the corresponding standards of knowledge, skills, and professional behaviours [31]. Since the EULAR-UEMS standards are based on the mapping of existing documents (from Europe and beyond) in rheumatology and other disciplines, they incorporate a wide range of perspectives and attitudes currently represented in medical training programmes. Furthermore, owing to the flexibility of its format and content, this document can be adapted to country/training centre-specific settings, in line with national/local regulations, while still ensuring the overarching goals are achieved. This document also provides guidance on the assessment methods and learning strategies for each of the 28 listed competences.

EULAR’s comprehensive and multimodal educational portfolio facilitates knowledge exchange, overcomes geographic barriers, and fosters international networking. Online courses and webinars are accessible to learners worldwide, including free-of-charge content for learners from limited-resource settings, and allow adaptation of learning pace to one’s schedule. On-site events such as live courses often include practical sessions providing young rheumatologists with tools to hone their skills, with the added value of peer-to-peer and peer-to-faculty networking. Over the years, EMEUNET has been actively involved throughout every phase of the above-mentioned projects, and this was instrumental in making the voice of European young rheumatologists heard, to identify their unmet needs and expectations, and ultimately to shape the future of postgraduate education in the EULAR area.

## THE TRAINING GAP: WHERE FORMAL PROGRAMMES FALL SHORT

Traditional training programmes, despite their comprehensive scope, cannot address all aspects of professional development required by young rheumatologists. Formal curricula typically focus on clinical competencies and knowledge acquisition but may inadequately address career development, research methodology, international collaboration skills, and adaptation to the evolving healthcare landscape.

Young rheumatologist networks have emerged to fill these gaps by providing peer-to-peer learning, mentorship opportunities, and professional development experiences that complement formal training. EMEUNET, PANLAR Joven, AYR, and the emerging YARN demonstrate how these networks can catalyse progress beyond traditional educational frameworks while respecting local contexts [27,32]. The collective experience of international young rheumatologist networks provides a foundation for catalysing global harmonisation efforts. Each network brings unique strengths: EMEUNET’s evidence-based educational initiatives, standardised assessment tools, creative workflows, and standard operating procedures [31], AYR’s large and diverse community, PANLAR Joven’s innovative virtual approaches, and YARN’s collaborative commitment. These networks, combined with EULAR’s demonstrated success in examination standardisation and competency framework development, provide replicable models for global harmonisation. The established relationships between continental networks, including partnerships with ACR, APLAR, PANLAR, and AFLAR, create opportunities for structured collaboration that transcends geographical boundaries [33]. EMEUNET’s experience in peer review mentoring programmes and research collaboration initiatives may offer frameworks that could support emerging networks, while learning from their innovative approaches to training challenges [34]. This bidirectional relationship can accelerate progress across all networks while maintaining respect for regional priorities and constraints.

A unique opportunity lies in establishing systematic cross-league mentor-mentee schemes connecting rheumatologists across continental boundaries, addressing the reality that rheumatology’s rapid evolution demands continuous professional development beyond initial specialist education [35,36]. These programmes would pair early-career professionals from emerging regions with established practitioners while facilitating reverse mentorship that brings fresh perspectives to experienced clinicians. Operating through virtual consultations, collaborative research projects, short-term exchanges, and digital grand rounds, such networks must address multiple career stages: early-career specialists transitioning to independent practice, mid-career professionals seeking subspecialisation, and senior practitioners exploring international collaboration. Mid-career mentorship programmes are a particular area with sparse support for future leaders, with occasional competitive fellowships like the National Institute for Health and Care Research’s specialty agnostic Future Leader and postdoctoral fellowship programmes for academics in the UK. Collaborative development of structured programmes for practice, research funding access, and teaching skill development, combined with flexible subspecialisation pathways that tap into virtual education models, can maximise shared resources across networks.

Contemporary rheumatology training must embrace alternative pathways that reflect global migration patterns and diverse career trajectories while integrating essential digital competencies as core rather than supplementary skills [[37], [38], [39]]. Rather than viewing migration as a zero-sum competition, collaborative networks can transform it into bidirectional knowledge transfer, supporting trainees wherever they establish careers while maintaining beneficial connections to regions of origin. Rheumatologists from resource-limited settings contribute valuable perspectives on tropical rheumatology and innovative problem-solving, while those migrating can maintain connections facilitating telemedicine consultations and collaborative research [40]. Simultaneously, networks must incorporate training in artificial intelligence applications, digital communication platforms, and social media engagement as fundamental capabilities, establishing shared standards for digital competency while respecting regional variations in technology adoption and accessibility.

## KEY SOLUTIONS AND LESSONS LEARNED

The ‘Rheumatology Training Around the Globe’ session at the 2025 EULAR Congress revealed both a growing convergence in rheumatology training structures and persistent regional disparities shaped by healthcare infrastructure and regulatory variations (Table). While regions like the US and parts of Europe benefit from standardised curricula and stable funding, many areas in Africa and Asia-Pacific face infrastructure limitations and trainer shortages. Nevertheless, several practical solutions emerged. Modular training frameworks, adapted from EULAR’s competency-based approach, allow flexible acquisition of recognised skills through clinical rotations, virtual learning, and short observerships. Digital learning platforms, exemplified by EULAR’s freely accessible online courses, provide scalable models now being adopted by PANLAR, APLAR, and African initiatives. Competency-based assessment, as defined by the EULAR-UEMS standards, shifts the focus from training duration to measurable outcomes, ensuring quality while allowing local adaptation. Peer support networks such as EMEUNET, PANLAR Joven, AYR, and YARN effectively address career development gaps and promote international collaboration beyond formal curricula. Finally, South-South and South-North partnerships, including RFA and UWEZO in Kenya, demonstrate how international collaboration can build sustainable local capacity without necessitating permanent relocation. Together, these initiatives offer realistic pathways towards more equitable and harmonised rheumatology training worldwide. All participating young rheumatology societies emphasised the unifying potential of peer support, digital learning, and sustained advocacy, creating a shared vision to harness international collaboration for global harmonisation while respecting local realities of each country.

This harmonisation does not represent homogenisation, but rather systems that celebrate diversity while ensuring excellence, accommodate local needs while maintaining global standards, and support individual advancement while strengthening collective capacity. Future harmonisation requires flexible, competency-driven frameworks adapted from successful models like EULAR-UEMS standards; expanded digital platforms with free or low-cost access for resource-limited settings; multilevel mentorship through formal young rheumatologist networks; sustainable investment in training infrastructure; and integration of national programmes into interconnected global networks.

Rheumatology’s future depends not on isolated excellence but on cultivating global connections and a shared commitment to improving worldwide patient care through collaborative innovation and mutual learning, transforming isolated national programmes into interconnected networks that strengthen the specialty through shared knowledge, resources, and collective expertise.

## CRediT authorship contribution statement

**Latika Gupta:** Writing – review & editing, Writing – original draft, Visualization, Supervision, Methodology, Data curation, Conceptualization. **Alexandros Panagiotopoulos:** Writing – review & editing, Writing – original draft, Visualization, Data curation. **Dzifa Dey:** Writing – review & editing, Writing – original draft. **Wilson Bautista-Molano:** Writing – review & editing, Writing – original draft. **Sunith Himantha Atukorale:** Writing – review & editing, Writing – original draft. **Ibtissam Gad:** Writing – review & editing, Writing – original draft. **Diego Benavent:** Writing – review & editing, Supervision, Methodology. **Tue Wenzel Kragstrup:** Writing – review & editing, Data curation, Conceptualization. **Alessia Alunno:** Writing – review & editing, Writing – original draft.

## Competing interests

LG leads the Asia Pacific League of Associations for Rheumatology (APLAR) Young Rheumatology Partnerships Committee and was ex chair of social media, Emerging EULAR NETwork (EMEUNET). AP serves as a member of the EMEUNET country liaison subcommittee. DD is the Past President of African League of Associations for Rheumatology (AFLAR) and Convenor of Young African Rheumatologists Network (YARN). WB-M is the Past Chair of PANLAR Joven. SHA is the past convenor of APLAR Young Rheumatologists (AYR). IG is the Past Chair of the American College of Rheumatology (ACR) Fellows-in-Training (FIT) Subcommittee and current member of ACR Committee of Education. DB is Chair of EMEUNET. TWK is the Past Chair of EMEUNET and convened the session at European Alliance of Associations for Rheumatology (EULAR) 2025. AA was the Chair of EMEUNET in 2018/2019. The views and opinions expressed are solely those of the authors and do not represent or reflect those of any affiliated institutions.

## Appendix Supplementary materials

Image, application 1

## Footnotes

Supplementary material associated with this article can be found, in the online version, at doi:10.1016/j.ero.2026.02.005.
