# Supplementary material for: Global perspectives on rheumatology training: insights from the EULAR 2025 EMEUNET Presents session
Source: EULAR Rheumatol Open. 2026 Mar 12;2(1):353–9. doi: 10.1016/j.ero.2026.02.005 (PMC13292126; doi:10.1016/j.ero.2026.02.005)
Supplement: Supplementary file 1 [file mmc1.docx]

**Supplementary Material**

**S1.** Africa: detailed structures, pathways, and partnerships

Structured training exists in Southern Africa, with several universities offering Health Professions Council-accredited subspecialty fellowships that include four-year comprehensive academic programmes and clinics (e.g., University of KwaZulu-Natal; University of the Witwatersrand) [1].

In West Africa, Nigerian and Ghanaian fellows can take the West African College of Physicians (WACP) and/or locally the Ghana College of Physicians (GCPS) and the National Postgraduate Medical College of Nigeria programs, which have harmonized curricula. These run on a two-to three-year, competence-based track. Francophone countries in sub-Saharan Africa, such as the Democratic Republic of Congo, Gabon, Togo, Senegal, Cameroon, and Ivory Coast, also have similarly structured programs. The rest have to train abroad or in other African countries, such as South Africa, with paediatric rheumatology capacity emerging from Nigeria, Kenya, and others [2].

**S2.** Asia‑Pacific: survey findings and programme characteristics

Assessment standards are rigorous, with qualification examinations required in all but one country. These typically combine written exams, clinical assessments, including objective structured clinical examinations (OSCEs) and viva voce, and board certification. Many programs require three-year logbooks documenting clinical exposure and research activities, some of which include dissertation requirements. Accreditation standards are mandatory in 14 countries, with one country requiring no accreditation.

Training duration averages six years post-medical school, comprising 3-4 years of pre-rheumatology training and 2-3 years of specialized training. Most countries (17/18) offer multiple training centres, providing broader access than that seen in Africa. Funding sources are diverse: government support (11 countries), training hospitals (6), and universities (3), with only one country providing no salaries. Data presented here, including training durations and funding patterns, draws from the APLAR Young Rheumatologists (AYR) member survey (unpublished data).

**S3.** Latin America and Pan‑America

Training structures lack standardization in most of the countries, with heterogeneous entry requirements, non-standardized core curricula, and varied assessment of practical skills and research knowledge. The typical pathway involves 2-3 years of internal medicine training followed by 2-3 years of rheumatology specialization, emphasizing clinical skills development but providing limited research exposure. While this clinical focus is valuable for patient care, it creates gaps in academic preparation and scientific methodology especially considering the limited time protected for research during training.

**S4.** United States

The standardized pathway requires completion of an undergraduate degree, medical school, internal medicine residency, followed by a competitive 2–3-year rheumatology fellowship. Rheumatology programs in the United States are accredited by organizations such as the Accreditation Council for Graduate Medical Education (ACGME), which outlines six core competency domains that are used to review trainee performance, and which include patient care, medical knowledge, system-based practice, practice-based learning and improvement, professionalism, and interpersonal communication skills [3]. To meet these competencies, training programs integrate comprehensive clinical exposure through inpatient consults, outpatient continuity and specialty clinics, and research exposure which may include basic and clinical research or quality improvement projects [3]. During training, rheumatology fellows enjoy protected time to ensure mastery of core curriculum through structured didactics like journal clubs, case discussions, and interdisciplinary conferences. Development of procedural competencies in musculoskeletal ultrasound, injections and aspirations are also integrated into the curriculum. Although many components of training are standardized, fellow exposure to opportunities like speciality clinics and ultrasound training are dependent on individual institutional resources and expertise. Fellows are formally assessed via in-training exams, written feedback, and annual reviews to monitor progress toward key milestones and in support of their professional development.

**S5. Europe**

The reported length of training programmes was up to 6 years (mean value 45 months). General internal medicine training, which was mandatory in 98% of the Countries, was performed prior to and/or during the rheumatology training programme with a mean reported length of 33 months. A final formal examination was reported in 33 out of 41 Countries [4].

**References**

1. Programmes Offered - Discipline of Rheumatology. https://rheumatology.ukzn.ac.za/programmes-offered/. Accessed 3 Sep 2025

2. Ines C, Hanene F, Ichrak K, et al (2022) 65 First francophone e-learning of pediatric rheumatology in Africa: the trainers’ opinion. Rheumatology 61:keac496.061

3. Acgme Rheumatology ©2024 Accreditation Council for Graduate Medical Education (ACGME) ACGME Program Requirements for Graduate Medical Education in Rheumatology.

Available from: https://www.acgme.org/globalassets/pfassets/programrequirements/2025-reformatted-requirements/150_rheumatology_2025_reformatted.pdf

4. Sivera F, Ramiro S, Cikes N, et al (2015) Differences and similarities in rheumatology specialty training programmes across European countries. Ann Rheum Dis 74:1183–1187
